# Understanding the Burden of Kidney Failure in Trinidad and Tobago: A Review of the Epidemiological Data From a Regional Center

**DOI:** 10.7759/cureus.40663

**Published:** 2023-06-19

**Authors:** Amit Ramrattan, Emile P Mohammed, Darren Bodkin

**Affiliations:** 1 Internal Medicine, Port of Spain General Hospital, Port of Spain, TTO; 2 Neonatology, UPMC (University of Pittsburgh Medical Center) Children's Hospital of Pittsburgh, Pittsburgh, USA

**Keywords:** non-communicable disease, epidemiology and public health, trinidad and tobago, caribbean science and public health, end stage renal disease (esrd)

## Abstract

Objective

The aim of this study was to determine the incidence of new patients requiring renal replacement therapy and to gather data on sex, age, ethnicity, mortality, and causes of kidney failure in Trinidad and Tobago in comparison with the rest of the world.

Method

Electronic data were gathered for new patients initiating dialysis between January 1, 2016, and December 31, 2017, including the date of dialysis initiation, age, gender, ethnicity, diagnosis, dialysis access and modality, and outcome at three months and the end of the year. The data were analyzed using simple descriptive statistics via Microsoft Excel (Microsoft Corporation, Redmond, Washington, United States).

Results

Over a two-year period, 265 new patients underwent renal replacement therapy, of which 51.7% were 50-69 years of age, 53.9% were male, 46% were female, 67.9% were Afro-Trinidadian, and 38.1% had a combination of diabetes mellitus and hypertension as the cause of kidney failure. The incidence rates of treated end-stage renal disease (ESRD) globally in 2016 and 2017 were 306 and 224 per million population, respectively, and mortality for both years was 32% and 32.1%, respectively.

Conclusion

Our study showed that Trinidad and Tobago has one of the highest incidences of patients initiating renal replacement therapy and mortality rates.

## Introduction

Chronic kidney disease (CKD) is a growing global health challenge with an increasing incidence that disproportionately affects developing countries [1]. Although the rates of end-stage renal disease (ESRD) in the Eastern Caribbean are thought to be high, there is a lack of epidemiological data available in the literature. This study aims to describe the incidence of patients requiring renal replacement therapy (RRT) in a regional hospital in Trinidad and Tobago, in addition to generating patient characteristics defined by age, sex, ethnicity, and comorbidities.

CKD is a matter of global scale, with the incidence of CKD requiring RRT almost doubling every 15 years [2], and Trinidad and Tobago is no exception. As the Port of Spain General Hospital (POSGH) is the only tertiary care hospital that serves the country’s capital city, it attracts most of the patients who require RRT from the region it serves. The hospital expanded its dialysis services over the last seven years from a four-station hemodialysis unit to a 12-station unit, with concurrent development of its peritoneal dialysis program to accommodate the growing number of patients.

Dialysis programs have significant financial implications with potentially devastating socioeconomic impacts, especially in developing countries without strong transplantation or prevention programs. Minimizing these socioeconomic impacts thus requires insight into the epidemiology of the disease.

## Materials and methods

Study design

This article presents a retrospective cohort study of new dialysis patients at the POSGH hemodialysis unit from January 1, 2016, to December 31, 2017.

Inclusion and exclusion criteria

We collected data on all new patients requiring RRT, whether they had acute or chronic conditions. Patients requiring RRT had an estimated glomerular filtrate rate (eGFR) of less than 15 ml/min that was established using the Modification of Diet in Renal Disease (MDRD) equation. No patients were excluded from this study, and patients with renal recovery did not have any repeated hospital admissions that required RRT. Renal recovery was defined as having an eGFR above 30 ml/min and not requiring RRT.

Data collection 

With the establishment of a new dialysis unit in August 2015, the North West Regional Health Authority (NWRHA) provided a computer for data collection purposes. All new patients who initiated dialysis at the unit between January 1, 2016, and December 31, 2017, were identified. A dedicated data-entry clerk, under the guidance of the renal unit staff and utilizing the available records, entered and stored the demographic information and data of each new dialysis patient on an Excel spreadsheet (Microsoft Corporation, Redmond, Washington, United States) for both 2016 and 2017. The collected data included details such as age, sex, ethnicity, comorbidities, mode of dialysis access, mode of dialysis initiation, and the date of the first dialysis session. The presence of comorbidities such as diabetes and hypertension was established prior to hospitalization and the commencement of RRT.

The outcome for each patient at three months and at the end of the year was then recorded. The outcome criteria included dialysis dependence, death within three months of dialysis, death after three months of dialysis, renal recovery within three months of dialysis, and renal recovery after three months of dialysis. At the end of each year (i.e., 2016 and 2017), the following data were compiled on two separate principal Excel spreadsheets: age, sex, ethnicity, comorbidities, dialysis access and modality, and outcome at three months and the end of each year.

Statistical analysis

Descriptive analyses were performed using the principal Excel spreadsheets by stratifying data by age, sex, ethnicity, comorbidities, and outcomes. The tabulated data were used to generate bar graphs, and percentages were calculated by dividing the tabulated data by 153 and 112, reflecting the number of new dialysis patients for the years 2016 and 2017, respectively. To estimate the incidence rate of new dialysis patients, the number of new cases was divided by 500,000 (to reflect the population catchment area) and multiplied by 1,000,000. Similarly, the mortality rate per 1000 person-years was calculated by dividing the number of deaths by the number of new cases and then multiplying by 1000.

## Results

The participant characteristics are shown in Table 1.

**Table 1:** Patient characteristics AKI, acute kidney injury; HIVAN, HIV-associated nephropathy; GN, glomerulonephritis.

	2016	2017	Overall Total (%)
Patients, n	153	112	265
Sex			
Male	84	59	143 (53.9)
Female	69	53	122 (46)
Age (in years)			
Mean	55 ± 14	59 ± 14	
Median	55	55	
Range	16-100	20-89	
Ethnicity			
Afro-Trinidadian	103	77	180 (67.9)
Indo-Trinidadian	26	20	46 (17.4)
Mixed	21	12	33 (12.5)
Chinese	1	2	3 (1.1)
Syrian	2	1	3 (1.1)
Comorbidities			
Diabetes and Hypertension	58	43	101 (38.1)
Diabetes	11	7	18 (6.8)
Hypertension	31	27	58 (21.9)
Vasculitis	12	2	14 (5.3)
Congential	1	0	1 (0.4)
Obstructive uropathy	16	6	22 (8.3)
AKI	6	7	13 (4.9)
HIVAN	5	6	11 (4.6)
Gout	2	0	2 (0.8)
Multiple myeloma	0	3	3 (1.1)
Primary GN	0	7	7 (2.6)
Unknown	11	4	15 (5.7)
Outcome			
Dialysis dependent	86	71	157 (59.2)
Died within 3 months of dialysis	38	23	61 (23)
Died after 3 months of dialysis	24	16	40 (15.1)
Renal recovery within 3 months of dialysis	13	4	17 (6.4)
Renal recovery after 3 months of dialysis	5	1	6 (2.3)

**Figure 1:**
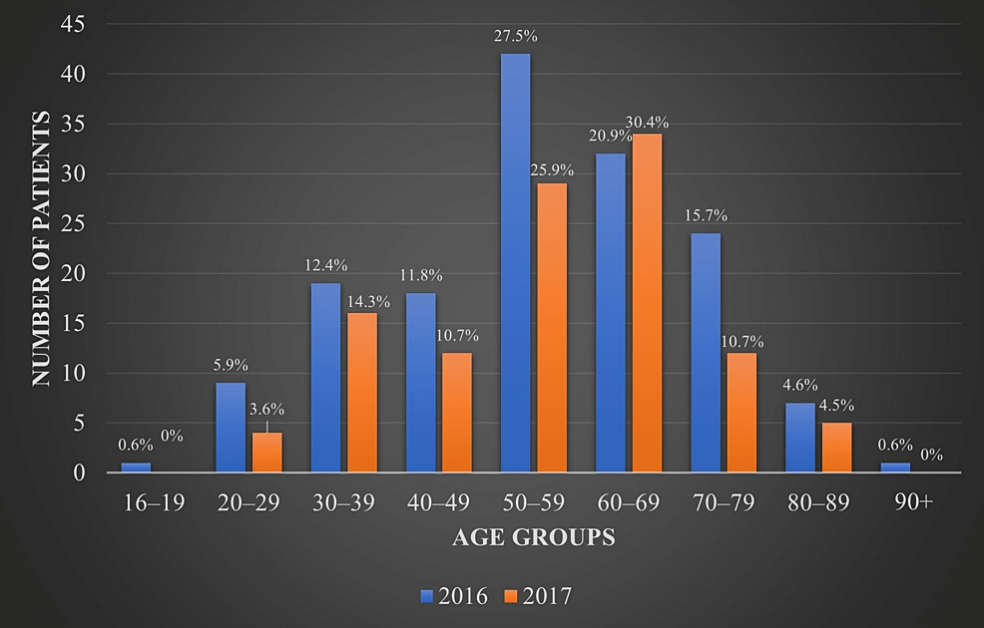
Age groups of patients on renal replacement therapy

In 2016 and 2017, 153 and 112 patients commenced RRT, respectively, with calculated incidence rates of 306 and 224 per million population (pmp). These rates were estimated considering that the NWRHA provides health services to a population of 500,000 [3]. In 2016, the most common age range was 50-59 years, accounting for 27.5% (42) of the cases, followed by the age group of 60-69 years at 20.9% (32), whereas the reverse was true for 2017, with 25.9% (29) of patients being in the age group of 50-59 years and 30.4% (34) falling into the 60-69 years age group. Overall, between 2016 and 2017, the majority of patients (51.7%) were between 50 and 69 years of age, but the ages ranged from 18 to 90 years, as seen in Figure 1.

**Figure 2:**
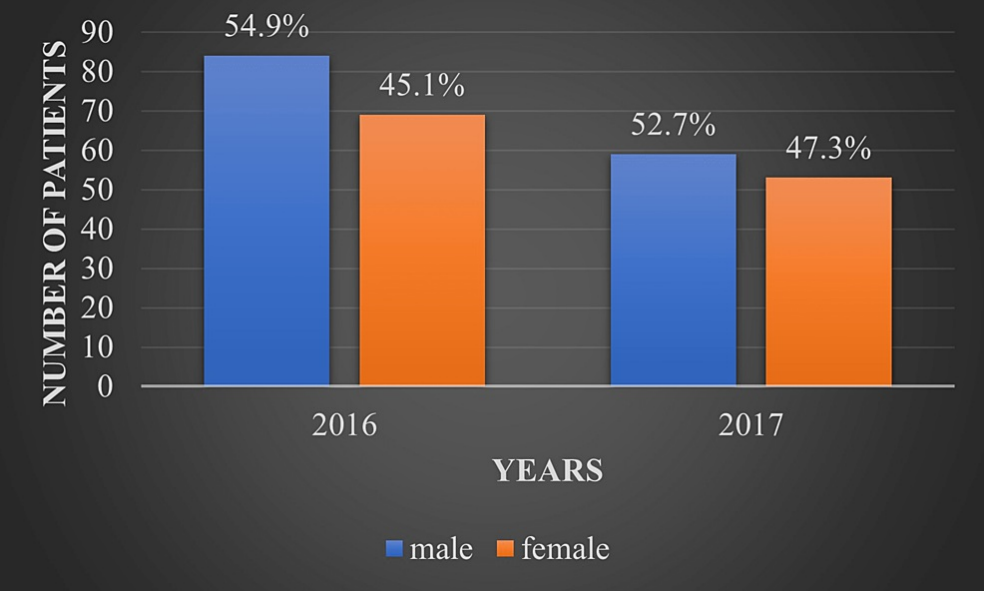
Sex of patients on renal replacement therapy

The sex ratio remained comparable for the years 2016 and 2017, with 45.1% (69) and 47.3% (53) of patients being female, while 54.9% (84) and 52.7% (59) of patients were male, respectively, as depicted in Figure 2.

**Figure 3:**
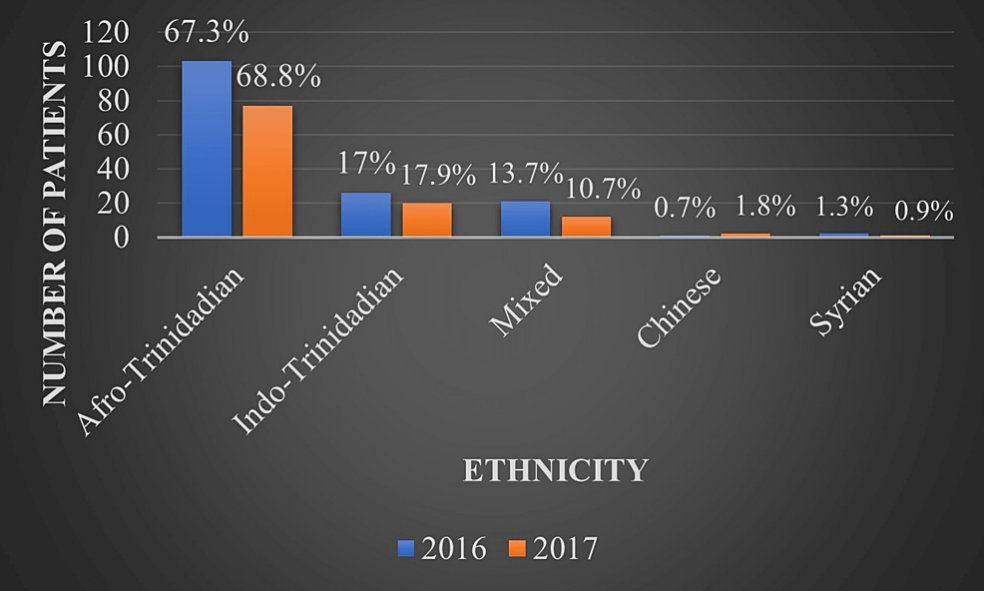
Ethnicity of patients on renal replacement therapy

The ethnicity breakdown of the patients was also similar for both years, with 67.3% and 68.8% of patients being Afro-Trinidadian and 17% and 17.9% being Indo-Trinidadian for 2016 and 2017, respectively. The third-most prevalent ethnicity was that of mixed race at 13.7% and 10.7% for 2016 and 2017, respectively, as seen in Figure 3.

**Figure 4:**
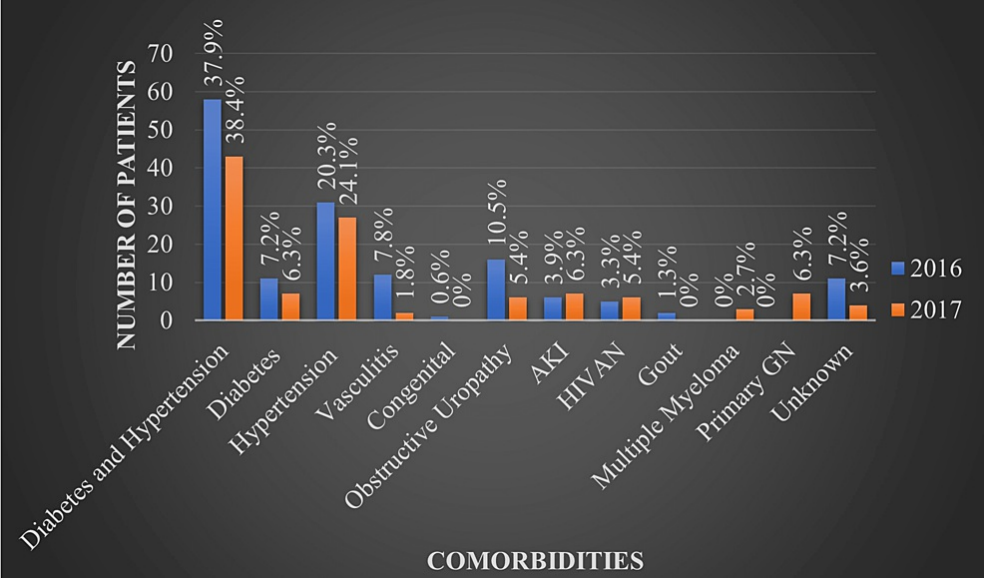
Comorbidities of patients on renal replacement therapy AKI, acute kidney injury; HIVAN, HIV-associated nephropathy; GN, glomerulonephritis.

The combination of diabetes and hypertension was the most common cause of CKD requiring RRT accounting for 37.9% and 38.4% of affected patients in 2016 and 2017, followed by isolated hypertension at 20.3% and 24.1%, respectively. Additionally, a significant number of other diagnoses were identified, including diabetes without hypertension (7.2% and 6.3%), obstructive uropathy (10.5% and 5.4%), vasculitis (7.8% and 1.8%), congenital (0.6% and 0%), acute kidney injury (AKI) (3.9% and 6.3%), HIV-associated nephropathy (HIVAN) (3.3% and 5.4%), multiple myeloma (0% and 2.7%), primary glomerulonephritis (0% and 6.3%), gouty nephropathy (1.3% and 0%), and unknown (7.2% and 3.6%) for the years 2016 and 2017, respectively, as seen in Figure 4.

Of the 153 new patients who received dialysis in 2016, 49 (32% or 320 per 1,000 person-years) died, 11 (7.2%) of whom died in less than three months following RRT commencement. Thirteen (8.5%) patients had recovered renal function within three months of their first dialysis, whereas five (3.3%) recovered outside of the three-month period. Eighty-six (56.2%) patients remained dialysis-dependent and alive after three months.

**Figure 5:**
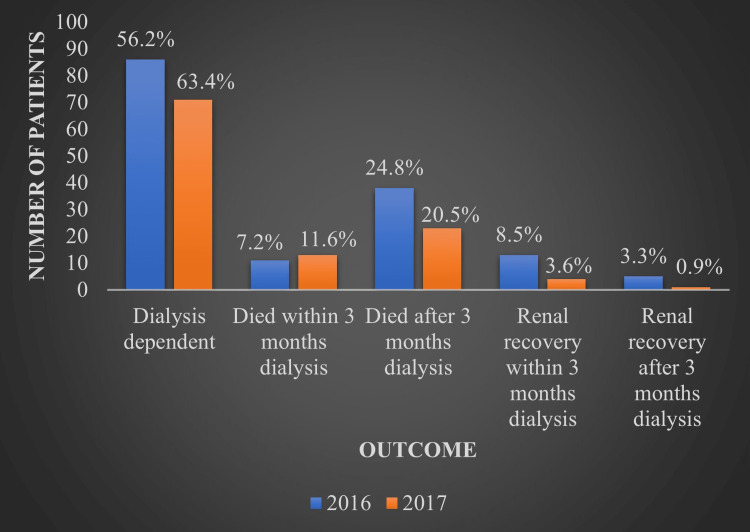
Outcome of patients on renal replacement therapy

Of the 112 new patients who received dialysis in 2017, 36 (32.1% or 321 per 1,000 person-years) died, 13 (11.6%) of whom died in less than three months following RRT commencement. Four (3.6%) patients had recovered renal function within three months of their first dialysis, one patient (0.9%) recovered outside of the three-month period, and 71 patients (63.4%) remained dialysis-dependent and alive after three months, as seen in Figure 5.

## Discussion

In its annual data report for 2020 [4], the United States Renal Data System (USRDS) highlighted the regions and countries with the highest incidence of treated ESRD (pmp) through the end of 2018, which included Jalisco, Mexico (594 pmp), Taiwan (523 pmp), Hungary (508 pmp), the United States (395 pmp), and Aguascalientes, Mexico (372 pmp) to mention a few. Prevalence exceeding 2000 pmp was seen in seven countries, five of which were in Asia. The highest prevalence of treated ESRD was in Taiwan (3587 pmp), Japan (2653 pmp), the United States (2354 pmp), Singapore (2255 pmp), Thailand (2028 pmp), Portugal (2014 pmp), and South Korea (2006 pmp). In the United States, the mortality rates of incident dialysis patients after the first year were found to be 294.8, 263.6, and 238 per thousand patient-years in 2004, 2009, and 2014, respectively.

Trinidad and Tobago was not included in the USRDS report, as the country had no national renal registry. As such data are paramount in planning for the many patients at risk for, and in need of, ESRD treatment, a hospital registry was established at the POSGH in an attempt to collect them. However, the POSGH was plagued with many challenges, including being damaged by an earthquake in August 2018, requiring a complete evacuation, which had halted the hospital’s ability to continuously collect data. Despite these challenges, the two-year period of complete data collection in 2016 and 2017 revealed high incidence rates of treated ESRD at 306 and 224 pmp, respectively, and with the mortality rate higher than that in the United States, one can speculate that Trinidad and Tobago has one of the highest mortality rates in the world.

Although Trinidad and Tobago lacks a national renal registry, an examination by the Administration of Special Health Care Programmes in the country estimated that as of May 2018, 1187 patients were receiving RRT [5]. This estimate likely corresponds to the study period of 2016-2017. With a population of 1,356,633 [6], this indicates a prevalence rate of 875 pmp. Such prevalence is concerning when compared to countries with similar incidence rates, as a high mortality rate is observed among the ESRD population in Trinidad and Tobago. Additionally, the examination highlighted that $12.9 million was allocated for dialysis patients in the fiscal year 2017, and an increase in numbers and costs is anticipated. This further emphasizes the economic burden associated with ESRD.

There are several other contributors to the high incidence rate of ESRD in Trinidad and Tobago, including the high prevalence of risk factors for CKD such as hypertension [7], diabetes [8], and obesity, in particular, an explosion of childhood obesity [9]. There are also very high rates of other risk factors such as autoimmune disease [10], prostate disease [11], presence of the APOL-1 gene [12], and low birth weights [13], but published data on their prevalence rates are lacking. This study highlights hypertension, diabetes, and obstructive uropathy being the leading causes of ESRD in Trinidad and Tobago. Over the two-year period, 177 (66.8%) of patients had either diabetes, hypertension, or both, as the cause for ESRD, suggesting deficiencies in primary care management. These conditions, if addressed and managed adequately, can undoubtedly decrease the incidence and prevalence of ESRD. A high mortality rate, where approximately one in three patients die annually, is a cause for concern. At an institutional level, medical practitioners must be vigilant in the management of these patients, and, at a national level, health leaders must implement measures to appropriately manage lifestyle comorbidities, which in turn can potentially be cost-effective, as a high incidence and prevalence of ESRD can lead to a financial strain.

This paper undoubtedly has limitations, and the incident rates illustrated may perhaps be an under-representation of the true national statistics. POSGH, despite providing healthcare services for a population of approximately 500,000, may not have captured all the patients requiring RRT, as some may have presented to private institutions within the catchment area. With respect to the comorbidities, the true number of patients with hypertension, vasculitis, or glomerulonephritis is likely inaccurate, as renal biopsy services were not available, and for the patients who presented with ESRD, a biopsy would not have altered the management or outcome.

The reasons for the disparity between the incidence and prevalence are complex but include limited access to RRT as well as high mortality rates of patients on RRT, which require further research. In the examination of the Administration of Special Health Care Programmes in Trinidad and Tobago report, a mention of 17 dialysis service centers was made, which include both public and private. When the public dialysis service is overburdened, patients transition into private centers funded by the government. This process, however, is intricate and requires assessment of the financial status of patients, which most times is low. A United Nations Development Programme report in 2022 [14] highlighted that 10,000 people lived in severe multidimensional poverty, and 56 thousand were vulnerable to multidimensional poverty. Further breakdown of these figures established deprivation to health and education. These barriers require further studies, but likely suggest limited access to primary care and RRT is not only a resource issue but one of limited health education and knowledge, which likely accounts for the high rates of diabetes, hypertension, and ESRD. There is also a fear of dialysis within society, thereby resulting in late presentations, with the majority of patients requiring acute dialysis in fact being late presentations of ESRD. Patients also often decline care or are not even referred for RRT. There are also complex environmental, educational, and cultural factors that negatively affect patients’ decisions to seek care early and to commence RRT in a timely fashion.

## Conclusions

This study has revealed that Trinidad and Tobago has one of the highest incidences and mortality rates of ESRD globally. There is an urgent need for the establishment of a national renal registry for the country, as it is necessary for strategic planning and research. Aggressive screening and prevention programs for CKD and health literacy programs must also be implemented as part of more robust non-communicable disease management programs, considering the projected increase in cases, which can lead to an economic burden.
